# Fitness to fly in the paediatric population, how to assess and advice

**DOI:** 10.1007/s00431-018-3119-9

**Published:** 2018-02-26

**Authors:** Joël Israëls, Ad F. Nagelkerke, Dick G. Markhorst, Marc van Heerde

**Affiliations:** 1 Department of Paediatrics, VU University Medical Center, De Boelelaan 1117, 1081 HV Amsterdam, The Netherlands; 2 Department of Paediatric Pulmonology, VU University Medical Center, Amsterdam, The Netherlands; 3 Department of Paediatric Intensive Care, VU University Medical Center, Amsterdam, The Netherlands

**Keywords:** Fit to fly, Air travel, Hypoxia, Trapped air, Neonate, Hypoxia altitude simulation testing

## Abstract

The number of children on commercial aircrafts is rising steeply and poses a need for their treating physicians to be aware of the physiologic effects and risks of air travel. The most important risk factors while flying are a decrease in partial oxygen pressure, expansion of trapped air volume, low cabin humidity, immobility, recirculation of air and limited options for medical emergencies. Because on-board medical emergencies mostly concern exacerbations of chronic disease, the medical history, stability of current disease and previous flight experience should be assessed before flight. If necessary, hypoxia altitude simulation testing can be performed to simulate the effects of in-flight hypoxia. Although the literature on paediatric safety of air travel is sparse, recommendations for many different situations can be given.

*Conclusion*: We present an overview of the most up to date recommendations to ensure the safety of children during flight.

**Table:** 

**What is Known:** • *Around 65% of on-board medical emergencies are complications of underlying disease*. • *In children, the three most common emergencies during flight concern respiratory, neurological and infectious disease.*
**What is New:** • *Although studies are scarce, some advices to ensure safe air travel can be given for most underlying medical conditions in children, based on physiology, studies in adults and expert opinions.* • *In former preterm infants without chronic lung disease, hypoxia altitude simulation testing to rule out in-flight desaturation is not recommended.*

**Electronic supplementary material:**

The online version of this article (10.1007/s00431-018-3119-9) contains supplementary material, which is available to authorized users.

## Introduction

Yearly, over one billion people travel by air and this number keeps growing. Likewise, the number of children on commercial aircrafts is rising [1]. Doctors are increasingly faced with the request to ascertain that it is safe to let their paediatric patient travel by air. This imposes a need for doctors to be aware of the physiologic effects of air travel and the risks of these effects in children.

The available data show estimations of medical incidents for all ages of 1.7 to 9.1 per 100,000 passengers [1, 2]. Around 10% of emergencies concern children, according to a single observational study [3]. Most emergencies (65%) are complications of underlying medical problems. In children, the three most common medical emergencies are infectious, neurological and respiratory disease [4, 5]. Only sparse literature on paediatric safety during air travel exists, which is why current guidelines are largely based on expert opinions. This article discusses the physiology of air travel and the assessment of fitness to fly in children with different underlying conditions and provides recommendations to travel safe with these conditions.

## Methods

Literature on air travel and children was searched for using Medline, Embase, Central and the Cochrane Database of Systematic Reviews in February 2017. No language restriction was used. Articles were selected if they (1) included children and (2) described air travel, hypoxia altitude simulation testing or altitude medicine. References of included articles were screened for relevant articles.

## Physiology of air travel

The main effect of increasing altitude is a decrease in barometric pressure (Pb). At sea level, the Pb is 760 mmHg which decreases by halve for every 5486 m (18,000 ft) of gained height. At high altitude, the percentage of oxygen remains stable at 21%, but with the drop in Pb, the partial oxygen pressure (pO_2_) decreases. Commercial airplanes cruise at approximately 11,582 m (38,000 ft), corresponding to a Pb of around 190 mmHg. The minimal allowed Pb in a pressurised cabin is approximately 564 mmHg, i.e. 74% of the Pb at sea level. With the decrease in Pb, the pO_2_ decreases from 160 to 119 mmHg, comparable to an oxygen percentage of 15% at sea level. To compensate for the decrease in pO_2_, the body adapts by increasing respiratory minute volume and cardiac output. The increase in alveolar ventilation causes a decrease in alveolar pCO_2_ and a rise in alveolar pO_2_ [6].

The other major effect of the decrease in Pb is the expansion of air volume. With a decrease in Pb of 74%, the volume expands up to 138%. This expansion affects all air trapped in an enclosed, gas filled, space in the human body. The change in volume is most rapid during ascent and descent. An explanation of altitude physiology in more detail is shown in Online Resource 1.

Besides the decrease in Pb, medical problems during flight can be provoked by low cabin humidity [7], prolonged immobility and recirculation of air with increased risk of transmitting infectious disease [1]. The concerns mentioned above are the risk factors to take into account when evaluating a child for fitness to fly. All are discussed in detail in the next paragraphs and summarised in Table 1.

**Table 1:** Risk factors for in-flight medical emergencies and advice on assessment in specific paediatric patient groups

Patient group	In flight risk factor	Risk of	Assessment	Advice
Anaemia	Low pO_2_	Syncope, decreased oxygen delivery	Hb	If Hb < 8.5 g/dL: transfusion or refrain from flying
Asthma	Limited medical care	Exacerbation	–	Emergency medication in cabin
Bronchopulmonary disease	Low pO_2_	Hypoxia	If < 1 year of age: HAST	SpO_2_ during HAST < 85%: recommend on-board oxygen SpO_2_ during HAST < 90%: consider on-board oxygen Currently receiving oxygen: double flow rate Current flow > 4 l/min: refrain from flying
Chronic pulmonary disease with long-term oxygen support (in previous 6 months)	Low pO_2_	Hypoxia	If no current oxygen support: HAST	SpO_2_ during HAST < 90%: recommend on-board oxygen Currently receiving flow: as for ‘bronchopulmonary disease’
Chest wall deformity	Low pO_2_	Hypoxia with insufficient compensation	Nightly ventilator support: HAST	Desaturation during HAST: on-board oxygen
Congenital heart disease	Low pO_2_	Hypoxia	Respiratory and circulatory status	Cyanotic heart disease: flying seems safe If NYHA class IV: refrain from flying or, if essential, on-board oxygen
Cystic fibrosis	Low pO_2_	Hypoxia	If FEV_1_ < 50%: HAST	SpO_2_ during HAST < 90%: recommend on-board oxygen
	Low humidity	Exacerbation	–	Consider extra dose of nebulised medication
Epilepsy	Limited medical care	Convulsion	–	Emergency medication in cabin
Immunodeficiency	Recirculation of air	Respiratory infection	–	Hand hygiene and, if possible, seating ≥ 2 rows from passengers with respiratory infection
Neonate, term	Low pO_2_	Apnea	–	Refrain from flying if < 1 week old
Neonate, preterm, no chronic lung disease	Low pO_2_	Apnea, hypoxia	Check for signs of respiratory infection	Refrain from flying until 3 months of corrected age. Lower respiratory tract infection: refrain from flying if < 6 months of corrected age
Neonate, preterm, chronic lung disease	Low pO_2_	Hypoxia	Respiratory status	Currently receiving oxygen: double flow rate Current flow > 4 l/min: refrain from flying
Neuromuscular disease	Low pO_2_	Hypoxia with insufficient compensation	Nightly ventilator support: HAST	SpO_2_ during HAST < 90%: recommend on-board oxygen
Otitis media	Pressure	Barotitis, pain	Otoscopy	Nasal decongestants, analgesics
Pneumonia	Low pO_2_	Hypoxia	Respiratory status, SpO_2_	Refrain from flying until afebrile, clinically stable and sPO_2_ ≥ 94% at sea level
Pneumothorax	Pressure	Tension pneumothorax	Chest x-ray	Refrain from flying until 7 days after resolution (14 days in case of trauma)
Pulmonary hypertension	Low pO_2_	Increase in pulmonary hypertension		Insufficient data. Consider on-board oxygen
Upper airway infection (recent)	Pressure	Barotitis	Otoscopy	Nasal decongestants. Chewing or sucking during ascent and descent
Sickle cell disease	Low pO_2,_ humidity	Veno-occlusive crisis		Adequate fluid intake, prevent cooling down
Thrombophilia (high-risk)	Immobility	Deep-venous thrombosis	–	Compression stockings
Trapped air, intrathoracic (e.g. cystic lung disease)	Pressure	Pneumothorax	If possible: determine size	Insufficient data for advice, discuss with specialist
Trapped air, intracranial (e.g. pneumocephalus)	Pressure	Intracranial herniation	If possible: determine amount of trapped air	Insufficient data for advice, discuss with specialist
Trapped air, in mechanical device (balloon, drain)	Pressure	Trauma	–	(Partially) deflate before ascent

*FEV1* forced expiratory volume in the first second, *HAST* hypoxia altitude simulation testing, *Hb* haemoglobin, *NYHA* New York Heart Association, *pO*_*2*_ partial oxygen pressure

## Medical assessment of fitness to fly in children

The medical history and, with chronic disease, timing of the most recent exacerbation are essential to check if any of the major concerns is relevant for the child. Previous flight experience and the estimated flight duration should be checked. Long flights above 6 h increase the risk of hypoxia [8]. The current respiratory state should be assessed. Pulse oximetry at rest and activity may show current hypoxia, but cannot rule out on-board hypoxia if normal. Both the British Thoracic Society (BTS) and Canadian Paediatric Society have guidelines which groups of patients should be evaluated before flight [1, 9].

A possible method to estimate the effect of a decrease in pO_2_ is the hypoxia altitude simulation test (HAST). A seated subject is placed in a whole body plethysmograph and given a mixture of 15% oxygen in nitrogen. In older children, a non-rebreathing mask can be used instead, although this is considered less reliable due to insufficient sealing of the mask [10, 11]. Supplemental oxygen during flight should be recommended if peripheral oxygen saturation (SpO_2_) falls < 85 or < 90% depending on age (< 1 or ≥ 1 year of age respectively) [9]. Safety of flying without on-board oxygen after a successful HAST has been validated for patients with cystic fibrosis and a forced expiratory volume in 1 second (FEV_1_) < 50% [12]. For young infants, studies show that HAST by means of a face mask does not adequately rule out in-flight desaturation [13]. Testing in a body plethysmograph is probably much more reliable, but to date, no study has included follow-up with measurements while flying [14]. If necessary, on-board oxygen can be safely administered by a portable oxygen concentrator (POC). There are strict regulations which POCs are allowed on board and differences exist between airlines. When planning air travel with a POC, adequate battery life, i.e. at least 150% of estimated travel time, should be ensured. The airline should be notified in advance [15]. The logistics of air travel with ventilator support are much more complicated. The airline should be noted in advance; necessary equipment should be available on board and a medical escort is required for ventilator-dependent patients [9].

## Hypoxia

The paediatric patients most at risk for the effects of hypoxia are (preterm) neonates, children with chronic or acute lung disease, anaemia, cardiac conditions and neuromuscular disorders.

Neonates show an immature response to hypoxia, which can induce apnea [16, 17]. Furthermore, they show a tendency for broncho- and vasoconstriction during hypoxia. These immature responses diminish in the first 2 months of life and are most pronounced in premature neonates until 36 weeks of gestation [17]. The clinical effects of a decrease in oxygen pressure have been studied in term and preterm neonates. Desaturation (SpO_2_ < 85%) during HAST is rare in term neonates during the first week of life but more common in preterm neonates, especially when diagnosed with bronchopulmonary dysplasia (BPD). In the latter, around 70% of neonates desaturate during HAST [11, 14, 18]. The recommendations of the BTS are to refrain from air travel for 1 week after birth in term children. Former preterm children without chronic lung disease are deemed fit to fly above 3 months of corrected gestational age [9]. Due to an increased risk of apnea, these infants should be advised to refrain from flying during a lower respiratory tract infection or significant upper respiratory tract infection, until 6 months of corrected age [9, 19]. Premature neonates with BPD should be thoroughly evaluated due to a high risk of desaturation during flight. In the first year of life, these former preterm children with BPD should be evaluated by HAST before flying to decide if supplemental oxygen is required during flight [9].

Children with chronic pulmonary disease (such as cystic fibrosis) should be evaluated, including spirometry when possible. With FEV_1_ < 50% or severe respiratory disease, HAST is advised to decide on supplemental oxygen while flying [9]. Patients currently receiving supplemental oxygen should have the flow rate doubled and refrain from flying if the flow rate at sea level exceeds 4 l/min. If children received long-term oxygen support up to 6 months ago, a HAST should be performed and in-flight oxygen available [9]. No evidence is available for children on long-term ventilation for pulmonary disease. Our expert opinion is to increase the percentage of delivered oxygen during flight to the maximum and refrain from changing the settings for pressure or volume. For patients receiving intermittent ventilator support, such as nightly support in certain neuromuscular conditions, a HAST should be performed and in-flight oxygen available [9].

In acute respiratory pathology, such as pneumonia, the advice is to refrain from flying until afebrile and clinically stable. Furthermore, there should be no additional oxygen requirement at sea level (oxygen saturation > 94%) [9].

For children with known anaemia, recent haemoglobin should be known to ensure the level is ≥ 8.5 g/dL (5.3 mmol/L) during flight. This value is what most airlines recommend, although no study to date has shown which levels of haemoglobin are associated with emergencies during flight. The intervention can be to cancel the flight or give a transfusion of erythrocytes [7].

The effects of hypoxia on underlying cardiac conditions in children are scarcely reported. The main risks are a further fall of SpO_2_ in cyanotic heart disease and a pulmonary hypertensive crisis. Two studies in patients with cyanotic heart disease show no clinically relevant hypoxia during flight [20, 21]. Data on adult patients with pulmonary hypertension show that clinically relevant desaturation occurs frequently during flight, mainly in more severe disease and with longer flight duration [22]. Safety of air travel cannot be adequately predicted with a HAST using a face mask in adults with pulmonary hypertension [23]. Cardiac patients should be evaluated by their specialist to decide if on-board oxygen is required. Patients with symptoms of heart failure at rest (NYHA class IV) are advised to refrain from flying. If nonetheless flying seems to be inevitable, on-board oxygen is advised [9].

Children with severe restrictive lung disease, caused by chest wall deformities or neuromuscular disease, might not be able to increase respiratory minute volume to compensate for the decrease in partial oxygen pressure. These patients should undergo a HAST to assess the need for on-board oxygen [24].

## Changes in air pressure

The paediatric patients most at risk for the effects of changes in air pressure are children with upper respiratory tract infections, recent pneumothorax and other forms of trapped air either intrathoracic, e.g. cystic lung disease, or extrathoracic, e.g. pneumocephalus.

The risks due to changes in air pressure follow from volume expansion. Assessment should include otoscopy to rule out otitis media. A recent history of air trapped in the thorax or skull, for instance after neurosurgery, requires imaging techniques to assess its volume.

The most frequent complication of changes in air pressure is pain and inflammation of the tympanic membrane, called ‘barotitis’. Studies report that 20% of children show otoscopic signs of barotitis after flight [25]. The main risk factor is a recent upper airway tract infection. To prevent barotitis, children are advised to chew, drink or suck during ascent and descent. After a recent upper airway tract infection, nasal decongestants are advisable, although the single relevant study shows little benefit for the prevention of barotitis in adults [26]. Current otitis media can lead to excess of pain during flight. Nasal decongestants, appropriate for age, and adequate analgesics are advised while on board [9].

Pneumothorax in a child is a contraindication for air travel due to the risk of developing a tension pneumothorax, which is described in case reports, usually with improvement of symptoms after landing [27]. The BTS advice is to confirm resolution of a pneumothorax with a chest x-ray and to refrain from flying until 7 days after resolution and 14 days in case of traumatic pneumothorax [9].

Other forms of trapped air, such as pneumocephalus after neurosurgery, can pose a health hazard. The consequences of a 138% increase in the volume of trapped air should be assessed and discussed with the appropriate specialist to decide whether the child should refrain from flying. Case reports have demonstrated that tension pneumocephalus can develop while flying, resulting in intracranial herniation and the need for emergency evacuation of trapped air [28]. A retrospective cohort study of 119 adults after craniotomy calculated that, based on computed tomography data, intracranial air volumes above 11 ml can result in intracranial hypertension during commercial flight [29]. Air trapped in a mechanical device, such as a cuffed tracheostomy tube, should be (partially) deflated to prevent trauma due to an increase in volume.

## Humidity and hydration

The paediatric patients most at risk for the effects of low humidity are children with cystic fibrosis and sickle cell disease.

In children with cystic fibrosis, the thick mucus in the airways can become even more dehydrated due to low cabin humidity, which in turn might exacerbate obstructive pulmonary disease. No data exist on the possible beneficial effects of nebulised medication before or during flight [12, 19].

In patients with sickle cell disease, the low air humidity with increased insensible losses, low fluid intake, cold and a decrease in partial oxygen pressure are all risk factors for a veno-occlusive crisis. According to some small studies, the risk of a crisis during flight is around 10%. The main advice is to prevent cooling down and ensure adequate fluid intake. The question whether these crises can be prevented with supplemental oxygen during flight has not yet been answered [30, 31].

## Infectious diseases

The paediatric patients most at risk for the effects of recirculation of air in a confined space are those with immunodeficiency. Furthermore, children with respiratory infections might spread the organism to other passengers.

To prevent airborne transmission of infectious disease, most aircrafts incorporate special filters and circulate the air from top to bottom which decreases the flow from passenger to passenger. These filters remove viruses that travel by means of large droplets and all bacteria and fungi [1]. The risk of acquiring an airborne illness is highest when sitting in the two rows nearest to an index case on flights above 8 h [32].

No current guidelines exist for children with immunodeficiency. Although the risk of transmission seems small, some preventive measures can be advised. The focus should be on good hand hygiene and, if possible, placing the child a minimum of two rows from a traveller with a current respiratory tract infection [33].

## Immobility

The main risk of immobility is the increased chance for a deep venous thrombosis (DVT). In general, the risk of developing DVT is much smaller in children than in adults, even with prothrombotic risk factors such as inherited thrombophilia [34]. Preventive measures to lower the risk of DVT are not indicated for the general paediatric population. No single study can be found to answer the question whether preventive measures are indicated in the case of additional risk factors, such as recent surgery, malignancy or inherited thrombophilia. The advice is to follow the most recent Cochrane review, based on studies in adults [35]. With moderately increased risk for DVT, such as inherited thrombophilia or obesity, compression stockings are advised, although the question remains whether this is feasible in children. For patients with the highest risk of DVT, such as a history of unprovoked DVT or active malignancy, a pre-flight prophylactic dose of low molecular heparin can be considered, especially for flights longer than 8 h. There is no evidence to support the use of acetylsalicylic acid to prevent DVT in adults and children. With active DVT, the advice is to refrain from flying for 4 weeks, or until the thrombosis has been treated and symptoms resolved [9].

## Medical emergencies during flight

Children with chronic disease should carry emergency medication with them. Asthma exacerbations are a common medical problem during flight, which emphasises the need to carry bronchodilators in the cabin with a suitable route of administration [9]. For patients with epilepsy, the risk of seizures is increased due to the combination of hypoxia, jet lag and fatigue [1].

Since 2004, all aircrafts are obliged to carry a medical flight kit for emergencies. These kits should contain, among other, dextrose, bronchodilators, epinephrine and antihistamines. Unfortunately, there is much difference between airlines in the contents of the medical flight kit and anticonvulsants, suitable for children, are not mandated [7, 36]. Most cabin crews are trained in adult cardiopulmonary resuscitation. In 75–85% of the on-board medical emergencies, a physician traveller responds to the call for help [4, 7].

## Conclusion and recommendations

The increasing number of children who travel by air stresses the need for their treating physicians to understand the physiology of air travel and the risks to their patients. Due to the fact that most medical problems during flight are exacerbations of known disease, the medical history and current clinical state are essential in deciding the fitness to fly of a child. This decision should consider the effects of hypoxia, changes in air pressure, low humidity, immobility, the risk of infectious disease and the limited options for handling medical emergencies. Additional testing, such as a hypoxia altitude simulation test, may be needed to assess the fitness to fly. Possible interventions follow from the specific condition and can include emergency medication, receiving on-board oxygen or to refrain from flying. Apart from the advice given in this article, airlines might have their own regulations on which patients are allowed on board and whether a physician signed fit to fly statement is mandatory. Current knowledge on paediatric safety during flight is limited. Most research has focused on (former) preterm neonates. For future research, we advise to use a body plethysmograph when performing a HAST and limit the use of face masks. Suggestions for future research are cut-off values for anaemia related to symptoms, necessity of preventive measures for DVT and how to treat patients on long-term respiratory support during flight.

## Electronic supplementary material

ESM 1 (PDF 776 kb)

## Abbreviations

BPD: Bronchopulmonary dysplasia
BTS: British Thoracic Society
DVT: Deep venous thrombosis
FEV1: Forced expiratory volume in the first second
HAST: Hypoxia altitude simulation testing
NYHA: New York Heart Association
Pb: Barometric pressure
pCO_2_: Partial carbon dioxide pressure
pO_2_: Partial oxygen pressure
POC: Portable oxygen concentrator
SpO_2_: Peripheral capillary oxygen saturation
