# Supplementary material for: Fitness to fly in the paediatric population, how to assess and advice
Source: Eur J Pediatr. 2018 Feb 26;177(5):633–9. doi: 10.1007/s00431-018-3119-9 (PMC5899119; doi:10.1007/s00431-018-3119-9)
Supplement: Supplementary file 1 — (PDF 776 kb) [file 431_2018_3119_MOESM1_ESM.pdf]

Fitness to fly in the paediatric population, how to asses and advice

European Journal of Pediatrics

Joël Israëls[1,2] (j.israels@vumc.nl), Ad F. Nagelkerke[2], Dick G. Markhorst[3], Marc van Heerde[3]

[1] Department of Paediatrics, VU University Medical Center, Amsterdam, The Netherlands
[2] Department of Paediatric Pulmonology, VU University Medical Center, Amsterdam, The Netherlands
[3] Department of Paediatric Intensive Care, VU University Medical Center, Amsterdam, The Netherlands

The physiology of altitude

When altitude increases, the barometric pressure decreases. This results in the expansion of air and ultimately a decrease in alveolar oxygen concentration. Figure 1 and table 1 sum up the effects of different heights (sea level, minimal allowed cabin pressure, summit of Mt. Everest and mean cruising altitude) on the barometric pressure (Pb), partial pressure of inspired oxygen (P_iO_2), expansion of trapped air and partial pressure of alveolar oxygen (P_AO_2).

Figure 1 Effect of height on barometric pressure, partial pressure of inspired O_2 and expansion of trapped air

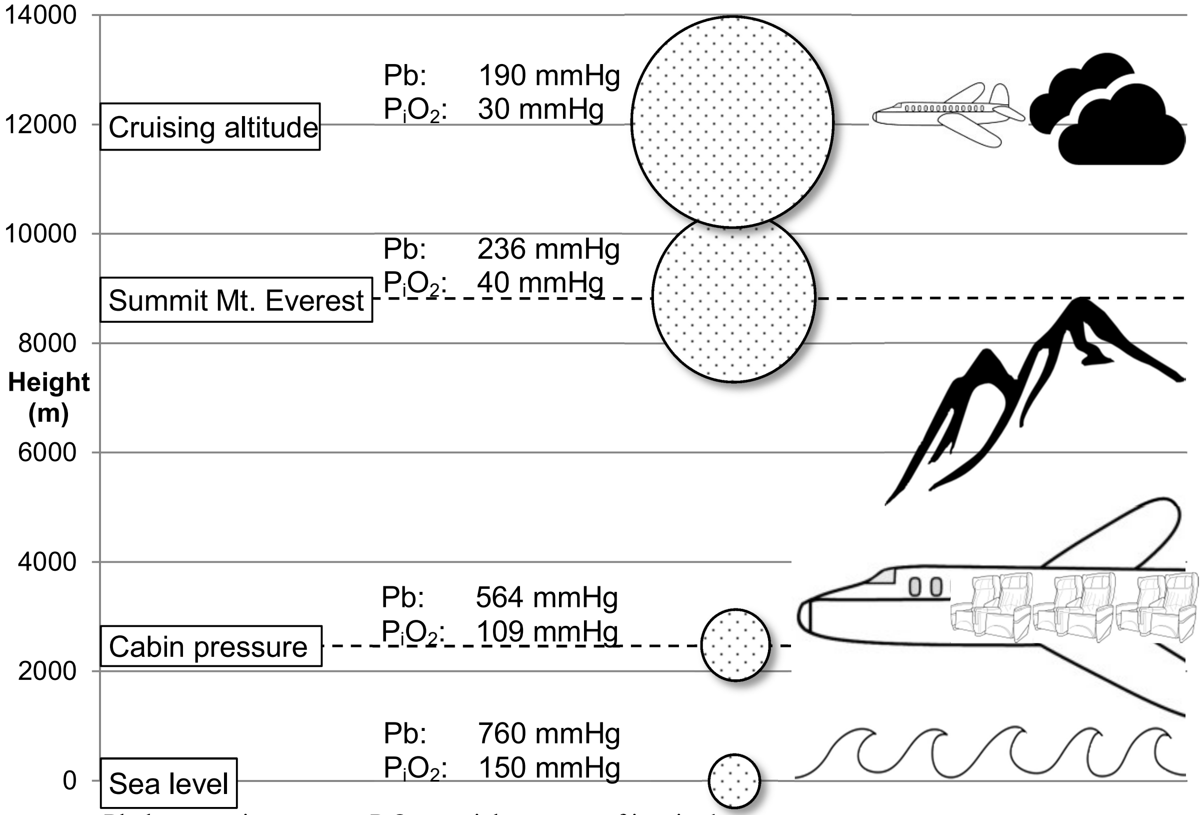

Pb, barometric pressure; P_iO_2, partial pressure of inspired oxygen

To understand the physiology of altitude a few physical principles are essential. The first is the inversely proportional relationship between pressure and volume at constant temperature (Boyle's law), which means that with a decrease in barometric pressure, the volume of air expands equally. The second is the fact that the total

pressure in a mixture of gasses is equal to the sum of the partial pressures (Dalton's law of partial pressures). The third is the alveolar gas equation: $P_AO_2 = 0.21(Pb\text{-}47) - P_ACO_2/R$, where 47 is the water vapour pressure in mmHg at 37 °C, P_ACO_2 is the alveolar partial pressure of carbon dioxide (around 40 mmHg with normal ventilation) and R the respiratory exchange ratio (the ratio between the elimination of carbon dioxide and the uptake of oxygen, which is 0.8 at rest) [1]. At sea level the barometric pressure is 760 mmHg and, in dry air, consists of 21% oxygen, 78% nitrogen and 1% other gasses. This composition is constant for the entire troposphere, the lowest layer of Earth's atmosphere, which reaches to 10980 m (36069 ft) at temperate latitudes [2]. With increasing altitude the Pb decreases by halve for every 5486 m (18000 ft) of gained height. At 2438 m (8000 ft), the Pb is 564 mmHg, which is the minimal allowed cabin pressure during air travel. This is 74% of the barometric pressure at sea level which, according to Boyle's law, leads to a 135% expansion of all air. With the decline in Pb and a constant percentage of oxygen, the partial oxygen pressure (pO_2) decreases equally. At sea level the pO_2 of dry air is 160 mmHg (21 % of 760 mmHg), which decreases to 119 mmHg at the minimal allowed cabin pressure (21 % of 564 mmHg). Taking into account a constant water vapour pressure of 47 mmHg, an alveolar partial carbon dioxide pressure of 40 mmHg and a respiratory exchange ratio of 0.8, the alveolar partial oxygen pressure decreases from 100 mmHg to 59 mmHg. To increase the amount of alveolar oxygen, as can be deducted from the alveolar gas equation, the body can decrease P_ACO_2 by increasing respiratory minute volume, i.e. hyperventilating [3].

Table 1 Effect of height on barometric pressure, partial pressure of inspired O_2 and partial pressure of alveolar O_2

**Height**			**Pb (mmHg)**	**P_iO_2**	**P_AO_2**
**Meter**	**Feet**			F_iO_2(Pb-47mmHg)	$P_iO_2 - P_ACO_2/R$
0	0	Sea level	760	150	100
2000	6562		596	115	65
2438	8000	Cabin pressure	564	109	59
5000	16404		405	75	25
8848	29029	Everest summit	236	40	0
12000	39370	Cruising altitude	190	30	0

Pb, barometric pressure; P_iO_2, partial pressure of inspired oxygen; P_AO_2, partial pressure of alveolar oxygen

References

1. West JB (2005) Respiratory Physiology: the essentials seventh edition. Lippincott Williams & Wilkins
2. Ahmedzai S, Balfour-Lynn IM, Bewick T et al (2011) Managing passengers with stable respiratory disease planning air travel: British Thoracic Society recommendations. Thorax 66 (suppl 1):i1-30. doi:10.1136/thoraxjnl-2011-200295
3. West JB, Schoene RB, Luks AM, Milledge JS (2012) High altitude medicine and physiology fifth edition. CRC Press
